# Molecular Characterization of Monoclonal Antibodies that Inhibit Acetylcholinesterase by Targeting the Peripheral Site and Backdoor Region

**DOI:** 10.1371/journal.pone.0077226

**Published:** 2013-10-11

**Authors:** Yves Bourne, Ludovic Renault, Sosthène Essono, Grégoire Mondielli, Patricia Lamourette, Didier Boquet, Jacques Grassi, Pascale Marchot

**Affiliations:** 1 Architecture et Fonction des Macromolécules Biologiques (AFMB), CNRS/Aix-Marseille Université, Campus Luminy, Marseille, France; 2 Ingénierie des Protéines, CNRS/Aix-Marseille Université, Faculté de Médecine - Secteur Nord, Marseille, France; 3 CEA, iBiTecS, Service de Pharmacologie et Immunologie (SPI), Laboratoire d’Etude et de Recherche en Immunoanalyse (LERI), Gif-sur-Yvette, France; 4 Centre de Recherche en Neurobiologie-Neurophysiologie de Marseille (CRN2M), CNRS/Aix-Marseille Université, Faculté de Médecine - Secteur Nord, Marseille, France; 5 CEA, iBiTecS, Service de Pharmacologie et Immunologie (SPI), Laboratoire d’Ingénierie des Anticorps pour la Santé (LIAS), Gif-sur-Yvette, France; Monash University, Australia

## Abstract

The inhibition properties and target sites of monoclonal antibodies (mAbs) Elec403, Elec408 and Elec410, generated against *Electrophorus electricus* acetylcholinesterase (AChE), have been defined previously using biochemical and mutagenesis approaches. Elec403 and Elec410, which bind competitively with each other and with the peptidic toxin inhibitor fasciculin, are directed toward distinctive albeit overlapping epitopes located at the AChE peripheral anionic site, which surrounds the entrance of the active site gorge. Elec408, which is not competitive with the other two mAbs nor fasciculin, targets a second epitope located in the backdoor region, distant from the gorge entrance. To characterize the molecular determinants dictating their binding site specificity, we cloned and sequenced the mAbs; generated antigen-binding fragments (Fab) retaining the parental inhibition properties; and explored their structure-function relationships using complementary x-ray crystallography, homology modeling and flexible docking approaches. Hypermutation of one Elec403 complementarity-determining region suggests occurrence of antigen-driven selection towards recognition of the AChE peripheral site. Comparative analysis of the 1.9Å-resolution structure of Fab408 and of theoretical models of its Fab403 and Fab410 congeners evidences distinctive surface topographies and anisotropic repartitions of charges, consistent with their respective target sites and inhibition properties. Finally, a validated, data-driven docking model of the Fab403-AChE complex suggests a mode of binding at the PAS that fully correlates with the functional data. This comprehensive study documents the molecular peculiarities of Fab403 and Fab410, as the largest peptidic inhibitors directed towards the peripheral site, and those of Fab408, as the first inhibitor directed toward the backdoor region of an AChE and a unique template for the design of new, specific modulators of AChE catalysis.

## Introduction

Acetylcholinesterase (AChE, EC 3.1.1.7) terminates cholinergic neurotransmission by rapidly catalyzing hydrolysis of the neurotransmitter, acetylcholine, at neuronal and neuromuscular synapses [1-3]. The active site of AChE, that contains the Glu/His/Ser catalytic triad and binds competitive reversible or irreversible inhibitors, is located at the center of the subunit at the end of a deep and narrow gorge [4]. At the enzyme surface and entrance of the active site gorge, the peripheral anionic site (PAS) encompasses overlapping binding loci for a range of reversible inhibitors and activators [5], and acetylcholine in certain conditions [6-8]. Inhibitor binding at the PAS appears to limit the catalytic rate by a combination of steric and electrostatic blockade of ligand trafficking through the gorge and by altering the active center conformation [9-12]. The molecular and electrostatics topographies and conformational flexibility of the PAS have been characterized thoroughly, but the mechanisms of its allosteric functioning to alter the active site geometry remain unclear [13-17].

Non-competitive inhibitors that bind the PAS of AChE include small organic cations such as propidium or gallamine [5,15,18], one quaternary group of bisquaternary inhibitors that fully occupy the gorge and bind both the active center and the PAS, such as decamethonium and BW284C51 [18-21], and the larger cation and first peptidic AChE inhibitor to be characterized, the three-fingered snake toxin fasciculin [13,22-26]. Crystal structures of fasciculin 2 (Fas2)-AChE complexes revealed the large surface area and multiple electrostatic and hydrophobic anchoring points solicited by the bound toxin at the PAS, along with apparent occlusion of the AChE gorge by the Fas2 central finger, loop II, all features being consistent with the nano- to picomolar affinity of the complex [13,26]. However, the structures failed to document the molecular or dynamical features responsible for the ~1% residual acetylcholine hydrolysis activity of the Fas2-AChE complex observed in solution [14,23-25]. Compatibility between the solution and structural data was suggested to require either conformational flexibility of the complex, creating a gap between the enzyme surface and the bound fasciculin, or opening of a backdoor, distinct from the gorge entrance, and whose transient enlargement would permit fractional substrate/product trafficking in the complex [13]. Shutter-like motion of the aromatic side chain of either residue Trp84 or residue Tyr442 (*Torpedo californica* AChE (TcAChE) numbering), which make thin walls between the active site pocket and the outside solvent in the putative backdoor region (BDR), have been visualized by molecular dynamics simulations [27-29] and x-ray crystallography [30,31], respectively.

In addition to fasciculins, various polyclonal and monoclonal antibodies (mAbs) have been shown to inhibit AChE by binding to “modulatory” sites on the enzyme surface (cf. References [S1-S15] in File S1). The target sites of three of them, raised against the *Electrophorus electricus* AChE (EeAChE) subunit and named Elec403, Elec408 and Elec410, were identified using complementary binding, inhibition and mutagenesis approaches [32,33]. In fact, Elec403 and Elec410 bind EeAChE competitively to each other and to Fas2, while only Elec403 also binds competitively with the organic PAS ligands propidium, decamethonium and BW284C51, and the substrate when in excess [32]. The Elec403- and Elec410-EeAChE complexes display residual activities of a few percent (i.e., slightly higher than that of the Fas2-AChE complex), while the Elec408-EeAChE complex displays one order of magnitude greater residual activity [32]. Inhibition of the residual activity of the Elec403 complex by the positively charged organophosphate inhibitor, echothiophate, and the transition state analog, TMTFA, occurs at lower rates than inhibition of the unbound enzyme, while for the Elec410 complex only inhibition by TMTFA is reduced, and for the Elec408 complex both inhibition rates are unaltered [33]. These features, along with delineation of the respective binding surfaces using mutagenesis of EeAChE [33], led to conclude that Elec403 and Elec410 are directed toward distinct but overlapping epitopes located at the enzyme PAS, whereas Elec408 targets a distinct regulatory site located in the BDR, and triggers a new inhibition mechanism. Finally, Elec403 and Elec408 are specific to EeAChE while Elec410 also inhibits, although to lesser extends, the venom enzymes from the snakes *Bungarus fasciatus* (BfAChE) and *Ophiophagus Hannah*, with IC_50_ values of ~1 nM and ~50 nM, respectively [32,34]. Hence, these three mAbs represent new promising peptidic tools to study allosteric regulation of AChE catalysis through surface binding sites.

To characterize the molecular determinants of these inhibitory “Elec” mAbs and provide structure-function relationships arguments into their binding site selectivity and mode of action, we have cloned and sequenced the three of them; generated, purified, characterized their antigen-binding fragments (Fabs); solved a crystal structure of Fab408 and designed homology models of Fab403 and Fab410; and used and validated a flexible docking approach to generate a model of a Fab403-EeAChE complex that tightly correlates with the available functional data. This complementary study documents, at the atomic level, the molecular peculiarities of new tools for exploring allosteric modulation of AChE catalysis: Fab403 and Fab410, as the largest peptidic ligands directed towards the PAS, and Fab408, as the first inhibitor directed toward the BDR. 

## Materials and Methods

### Cloning and sequencing of the Fabs

The three mAbs were produced as IgG1,κ from murine hybridomas [32]. The cDNAs corresponding to the variable regions in the heavy and light chains (VH and VL) were obtained using the specific Cγ1 primer (5′-AGG GGC CAG TGG ATA GAC-3′), complementary to the sequence encoding residues 141–147 of the CH region of the γ1 chain, and the RevCκSal 1 primer (5′-GCT GAT GCT GCA CCA ACT AWG TCC ATC TTC-C3′), complementary to the sequence encoding residues 209–214 of the CL region of the κ chain [35]. The mixture containing 7 μl of RNA (~5 μg), 1 μl of dNTP (final concentration, 500 μM), 1 μl of primers (final concentration of each, 0.2 μM), and 7.5 μl of RNase-free water was heated at 70°C for 10 min. Primer annealing and reverse transcription were carried out in the presence of 1 μl of AMV reverse transcriptase (1 U/μl) and 0.5 μl of RNase inhibitor (1 U/μl) at 42 °C for 50 min. The final 20 μl sample containing the cDNA products was stored at −20°C until use. PCR amplification of the VH and VL sequences used primer mixtures as described [36]. In brief, the final 50 μl sample, containing 5 μl of first-strand cDNA, 0.2 μM of back and forward primers, 200 μM of dNTP, 1.5-5.5 mM of MgCl_2_, and 5 μl of 10x buffer, was heated at 92°C for 3 min, and then subjected to 25 cycles (92°C for 1 min, 72°C for 30 s, 72°C for 1 min) in presence of 1 unit of DyNAzyme™ EXT DNA Polymerase (Finnzymes, Oy, Finland). The reaction was stopped with a 72°C step for 7 min. One-tenth of each PCR reaction was analyzed on a 2% agarose gel stained with ethidium bromide. The sequences of the PCR products were verified (Eurofins MWG Operon), and analyzed using the IMGT/V-QUEST tool [37,38]. The nucleotide sequences have been deposited with The EMBL Nucleotide Sequence Database, accession codes HE984312 and HE984313, HE984314 and HE984315, and HE984316 and HE984317 for the VL and VH domains of Elec403, Elec408 and Elec410, respectively. 

**Figure 1:**
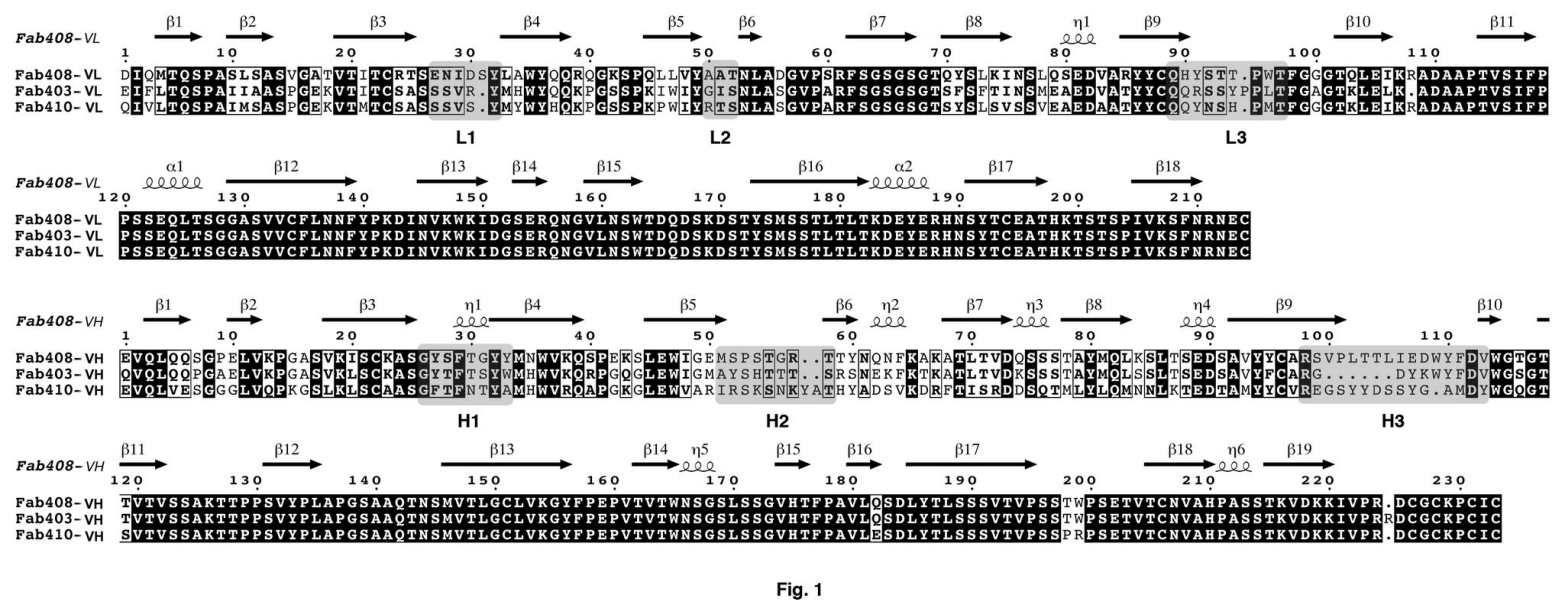
Sequences and numbering of the three anti-EeAChE Fabs. The light (L, top) and heavy (H, bottom) chains are displayed. The residue numbering and secondary structure elements displayed above the alignment are those of Fab408. Conserved residues are shown on a *black* background and non-conserved residues on a *white* background. The CDRs, defined according to http://www.bioinf.org.uk/abs/ (cf. Figure S1 in File S1), are highlighted as grey boxes and labeled.

The Fab complementary determining region (CDR) boundaries were defined according to the IMGT standards (Figure S1 in File S1). However, to avoid virtual gaps in the structure coordinates and inconsistencies related to definition of β-strands, the consecutive numbering of residues and Greek letter labeling of β-strands and α-helices will be used herein (Figure 1).

### Biochemical and functional analyses

Protein purification and preparation, biochemical and functional analyses, and N-linked carbohydrate removal used standard procedures as described as Supplemental Experimental Procedures in File S1.

### Solution and refinement of the Fab408 structure

The procedures for crystallization of Fab408 and data collection are described as Supplemental Experimental Procedures in File S1. Initial phases were obtained by molecular replacement with the AMoRe program package [39] using, as search models, the structures of the variable and constant domains of Fv Huh52-Aa (accession code 1FGV) and FabE8 (accession code 1WEJ), respectively. This procedure yielded a correlation coefficient of 53.9% and an R-factor value of 39.1% in the 10-4 Å resolution range. Automatic building of the initial model with ARP/wARP yielded a virtually complete model consisting of a single Fab408 molecule [40]. The model was improved by manual adjustment with the graphics program COOT [41] and was refined with Refmac [42] including TLS refinement with each variable and constant domain defining a TLS group. Data collection and refinement statistics are summarized in Table S1 in File S1.

The final structure of Fab408 comprises 213 and 224 residues for the L and H chains, respectively, and 208 water molecules. CDR-L1 adopts a conformation similar to the canonical structural class 2 [43,44], CDR-L2, CDR-L3 and CDR-H1 belong to class 1, CDR-H2 belongs to class 2 and CDR-H3 does not belong to an identified class. High temperature factors and weak electron densities are associated with segments Glu42-Lys43, Thr55-Thr58, Lys65-Lys67, Leu105-Trp109 and Gly138-Ser145 in the H chain and residues Asn212-Ala213 in the L chain (Figure 1). Stereochemistry of the structure was analyzed with MOLPROBITY [45]; only L chain residue Ala51 was found in the disallowed regions of the Ramachandran plot. Six *cis*-Pro residues are found at positions 158, 160 and 200 in the CH domain and at positions 8 and 95 in the VL domain and 141 in the CL domain; all six positions are conserved in the three Fabs (Figure 1). The atomic coordinates and structure factors of Fab408 have been deposited with the RCSB Data Bank, accession code 2YMX.

### Theoretical modeling of Fab403 and Fab410 and of a Fab403-EeAChE complex

The Fab403 and Fab410 models were built using MODELLER [46] and, as a template, the structure of the Fab fragment of the virus-neutralizing mAb SD6 (accession code, 1QGC, 77-80% identity), selected using the TM-score from the HHpred server [47]. The average root mean square deviation values between the Fab403 and Fab410 models and the template are ~0.34 Å and ~0.25 Å, respectively, for 212/213 Cα atoms. Between the three Fabs the values are in the 1-2 Å range for 212 Cα atoms, with the highest deviations observed for the solvent-exposed CDR-H2 and CDR-H3 of Fab403 and Fab410. In the Fab403 model, CDR-H1, CDR-H2, CDR-L1, CDR-L2 belong to structural classes 1, 2, 1, 1, while CDR-H3 and CDR-L3 do not belong to an identified class; the side chains of CDR-H1 residues Thr28, Thr30-Trp33; CDR-H2 residues Tyr52, His54, Thr55, Thr57; CDR-H3 residues Arg98, Asp100-Trp103, Asp106; CDR-L1 residues Ser26-Ser28, Arg30, Tyr31; CDR-L2 residues Ser51 and Asn52; and CDR-L3 residues Arg90, Ser91, Tyr93, point towards the solvent. In the Fab410 model, CDR-L1, CDR-L2, CDR-L3, and CDR-H1 belong to class 1 and CDR-H2 to class 4; CDR-H3 does not belong to an identified class; the side chains of CDR-L1 residues Ser27, Ser28, Ser30, Tyr31; CDR-L2 residues Arg49, Ser51; CDR-L3 residues Tyr90, Asn91, His93; CDR-H1 residues Thr28, Asn30-Tyr32; CDR-H2 residues Arg52, Lys54, Lys57, Tyr58; and CDR-H3 residues Arg100, Ser103-Ser108, point towards the solvent.

A model of the EeAChE subunit, devoid of the EeAChE-specific insert Ile418-Gln446 and C-terminal T peptide Ala576-Leu610 (Figure S2 in File S1) [48], was designed using the structure of a TcAChE subunit (accession code 1EA5) as a template and the same procedure as above described for the Fab models. The average root mean square deviation value between the model and template is 0.18 Å for 532 Cα residues.

The model of the Fab403-EeAChE complex was generated by flexible docking using HADDOCK 2.1 [49] and default parameters and, as possible interfacial active residues, nine residues of the Fab403 CDRs (four from the H chain and five from the L chain) and the three interacting Ser75, Gln279 and Leu282 residues of EeAChE [33]. Neighboring solvent-accessible residues (four for Fab403 and four for EeAChE, including the potentially N-glycosylated Asn345) suitably positioned for being indirectly involved in the binding, were defined as passive residues. For each run, the top 200 complexes generated after rigid body energy minimization were subjected to flexible simulated annealing in torsion angle space and to flexible water refinement in Cartesian space, and the three energetically best models scored by HADDOCK were comparatively analyzed.

The models of the Fas2-mouse AChE (mAChE) and Fas2-EeAChE complexes were generated using a similar approach and a randomly oriented Fas2 molecule relative to the mAChE (accession code, 1KU6) and EeAChE subunits. Similar ambiguous interaction restraints were defined from each partner corresponding to five and four active residues from the interfacial regions of mAChE/EeAChE (PAS region) and Fas2 (loop II), respectively, associated to three surrounding passive residues from each partner. 

### Structural analysis and comparisons

Electrostatic surface potentials of the three Fabs and of EeAChE, TcAChE, and mAChE were calculated using APBS [50] with the PyMOL APBS tools. The molecular surface area buried to a 1.4 Å radius probe at the Fab403-EeAChE complex interface was calculated using Areaimol [51]. Structural superimposition of this complex with the Fas2-mAChE (accession code 1KU6 [13]), BW284C51-TcAChE (1E3Q [21]), decamethonium-TcAChE (1ACL [20]), propidium-mAChE (1N5R [15]), and ACh-mAChE (Ser203Ala mutant) complexes (2HA4 [7]), based on the respective AChE subunits, used COOT (root mean square deviation values, 0.89-0.98 Å for 521-526 Cα atoms). Figures were generated using PyMOL [52].

## Results and Discussion

### Cloning and sequencing of mAbs Elec403, Elec408 and Elec410

Analysis of several independent clones resulted in unambiguous consensus nucleotide sequences for the VL and VH domains of each of the three mAbs (Figure 1A). Homogeneity in the results validated the cloning strategy, while the amino acid sequences of the Elec408 VL and VH domains were further validated during refinement of the crystal structure. All VL and VH regions are associated with a germline V region allele, and it is likely that the observed mutations were induced by the affinity maturation process associated with the repeated immunization protocols used to generate the mAbs [32]. The sequences of these antibodies reveal an abundance of Tyr residues in the CDRs that, combined with flexible Ser/Gly residues, reflect functional paratopes capable of mediating antigen recognition with high affinity and specificity [53]. The observed level of mutation throughout the three mAbs is generally classical [54], except for Elec403 whose CDR-H2 and subsequent two residues are entirely modified (Figure S1A in File S1). In fact, close homology of the new sequence with that of Fas2 loop I, which binds at the periphery of the gorge entrance in the Fas2-AChE complex [13,26], is intriguing. Even though similar sequences, either straight or reverse, can be found in unrelated proteins, hypermutation of this CDR into this sequence may denote particular adaptation pressure toward the EeAChE PAS. 

### Physicochemical and functional characterization of the Fabs

**Figure 2:**
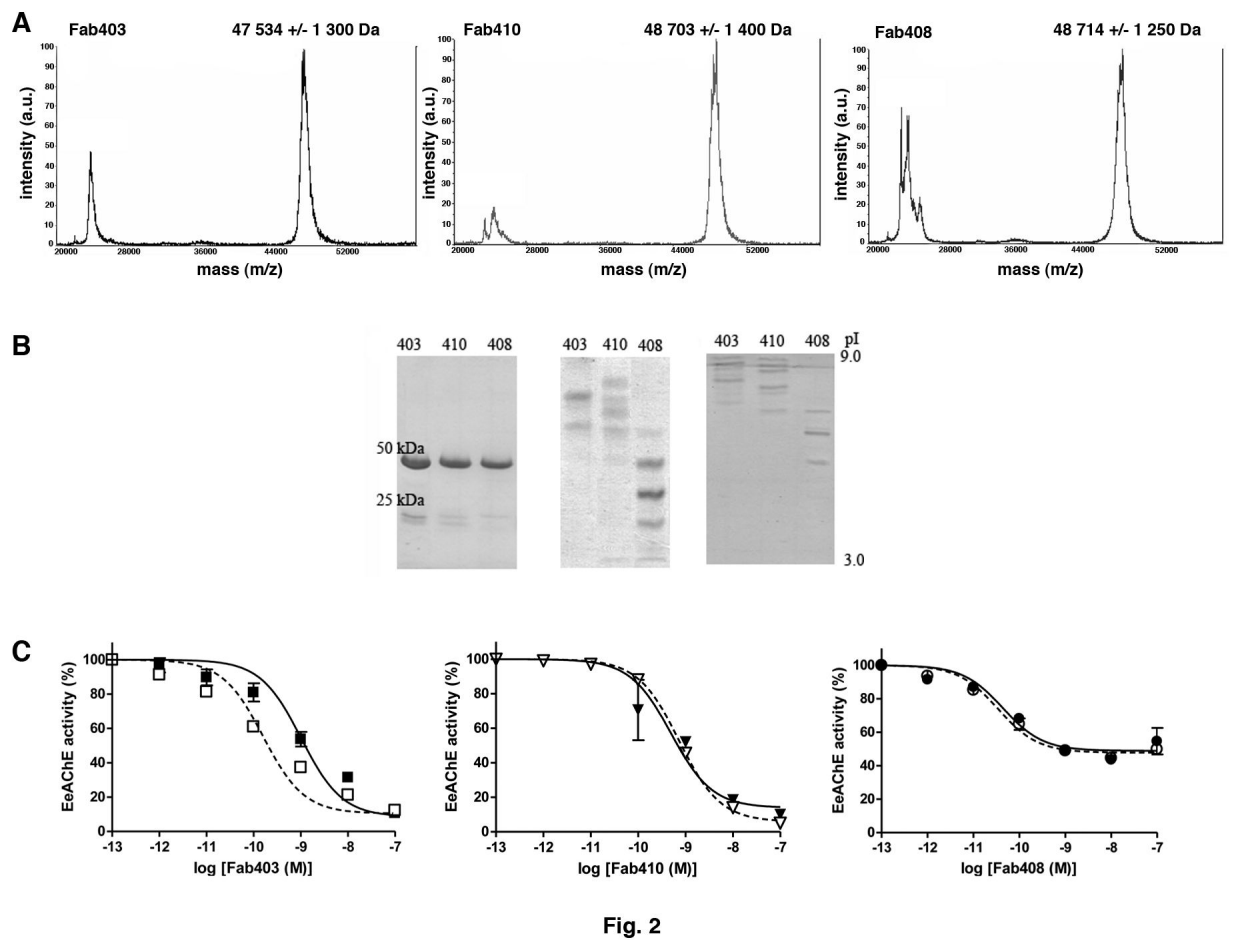
Physical and functional characterization of the purified Fabs (typical experiments). (**A**) MALDI-TOF mass spectrometry profiles, showing both the di-charged and mono-charged entities. Note the satisfactory homogeneity in mass of the latter. (**B**) Electrophoresis patterns obtained by SDS-PAGE *in* *non-reducing* *conditions* (12.5% PhastGel, left) and native-PAGE (7.5% PhastGel, center) with migration from the cathode (top) toward the anode (bottom), and by isoelectric focusing (pI 3-9 PhastGel, right). The three Fabs are more homogenous in mass than in charge, a feature that likely result from variations in the C-terminus generated by papaine cleavage of the CH chain; yet, all isoforms bind EeAChE equally, as verified by a native-PAGE mobility shift assay (not shown). The neutral average pI of Fab408 and cationic average pIs of Fab403 and Fab410 are evident. (**C**) Inhibition of native EeAChE (closed symbols, full lines) and N-deglycosylated EeAChE (open symbols, dashed lines) by the three Fabs. The higher residual activity recorded at saturating concentration of Fab408, compared with Fab403 and Fab410, is evident. Removal of the six N-linked glycan chains of EeAChE results in 6.5-fold higher affinity for Fab403 but unaltered residual activity at saturating Fab403 concentration. Data points correspond to the average values of duplicates or mean values of triplicates. Non-linear fitting of the data points used a sigmoidal equation. Mean values of the mass, pI, IC50 and residual activity of each Fab are reported in Table 1.

Fab403, Fab408 and Fab410 were generated from the parental IgGs by cleavage of the H chains, purified using standard procedures and analyzed using complementary techniques (Figure 2AB; Table 1). MALDI-TOF mass spectrometry and SDS-PAGE analyses showed proper molecular masses, of *ca.* 50 kDa, and high homogeneity apart from minor scattering and traces of unlinked H and L chains. Native-PAGE analysis revealed well-defined bands indicative of Fab isoforms differing in their net charges, along with greater average mobility towards the anode for Fab408 compared to Fab403 and Fab410. Isoelectric focusing analysis showed similar migration patterns to native-PAGE and led to define average pI values of ~9.0 for Fab403, ~7.0 for Fab408 and ~8.5 for Fab410, similar to the theoretical values calculated from the sequences. These values are also consistent with binding of Elec403 and Elec410, but not Elec408, to the PAS of EeAChE, as do the cationic inhibitors Fas2, propidium and gallamine [15,32,33]. 

**Table 1:** Biochemical and functional parameters for the purified Fabs and their EeAChE complexes.

**Fab**	**Fab403**	**Fab408**	**Fab410**
Theoretical mass (kDa) ^a^	46.8	47.5	48.5
Monoisotopic mass (kDa) ^b^	47.5 ± 1.3	48.7 ± 1.2	48.7 ± 1.4
Theoretical pI **^a^**	8.6	6.3	8.1
Observed pI **^c^**	9.0	7.0	8.5
**Enzyme**	**EeAChE**	**EeAChE**	**EeAChE**
Binding site	PAS	BDR	PAS
IC_50_ IgG (nM) ^d^,^f^	(1)	(0.04)	(0.04)
IC_50_ Fab (nM) ^d^	5.1 ± 2.38	0.14 ± 0.06	0.53 ± 0.24
k_i_ Fab (nM^-1^min^-1^)	0.016 ± 0.023**^e^**	0.255 ± 0.061**^d^**	0.029 ± 0.015**^d^**
k_-i_ Fab (min^-1^)	0.075 ± 0.019**^e^**	0.015 ± 0.009**^d^**	0.033 ± 0.004**^e^**
Calculated K^i^ Fab (nM)	4.64 ± 0.42	0.06 ± 0.07	1.13 ± 0.02
Residual activity Fab (%) ^d^	6.6 ± 1.9	30.0 ± 2.6	4.7 ± 1.0
Residual activity IgG (%) ^d^ ^,^ ^f^	(3)	(30)	(6)

a Calculated from http://web.expasy.org/protparam/

b By MALDI-TOF mass spectrometry

c By isoelectric focusing

d Values shown as means ± SD (n > 2)

e Values shown as averages ± the variation (n = 2)

f Values in parentheses are from [32]

nd, not determined

Functional analysis of the three Fabs addressed their capacity to bind to and inhibit the EeAChE tetramer enzymatically processed from the natural asymmetric dodecamer [32,55] (cf. Reference [S5] and Supplemental Experimental Procedures in File S1). For each Fab, all charge isoforms were found to bind EeAChE equally, as assessed by a native-PAGE mobility shift assay (data not shown). As well, both native-PAGE mobility shift and AChE inhibition assays assessed for mutually exclusive binding of Fab403 and Fab410 to EeAChE, while either could bind simultaneously with Fab408 to form stable ternary complexes of two different Fabs per EeAChE subunit (data not shown). Upon equilibrium binding analysis, IC_50_ values in the 10^-10^-10^-9^ M range, and values of residual activity at saturating Fab concentrations in the 4-7% range for Fab403 and Fab410 and of ~30% for Fab408, were obtained (Figure 2C; Table 1), similar to the values reported for the parental IgGs [32]. Kinetic analyses revealed 2-fold slower association (ki constant) and ~2-fold faster dissociation (k-i) of Fab403 compared with Fab410 (Table 1), suggesting different levels of conformational adaptation of their combining sites to their binding subsites at the EeAChE PAS. In turn, the Fab408 association rate, one order of magnitude greater than those of its two congeners, and its dissociation rate, 5-fold and 2-fold lower respectively, may denote genuine complementarity of the Fab408 combining site with its binding surface in the BDR. In all three cases the calculated dissociation constants (Ki) match the IC50 values. The reverse ranking in the Ki values, which reflect the Fab403/Fab410 and Fab408 capacities to bind the EeAChE surface, relative to the residual activities, which reflect the Fab capacities to inhibit the EeAChE activity, is consistent with the PAS and the BDR triggering distinctive mechanisms for allosteric inhibition of catalysis. 

### Effect of N-linked carbohydrate removal on EeAChE activity and Fab binding

There are six consensus sites for N-glycosylation at the surface of the EeAChE subunit (Figure S2 in File S1). PNGaseF treatment of EeAChE in native conditions followed by SDS-PAGE analysis resulted in lower apparent molecular masses for each of the three bands, of *ca.* 80, 50 and 30 kDa, typically observed for the reduced subunit (cf. Figure 1 in [55]) (data not shown). The positional shift was identical to that observed with a fully deglycosylated EeAChE control, thereby assessing for optimal removal of all PNGase-sensible glycan chains despite the milder conditions. In contrast, native-PAGE showed no significant migration difference between the native and deglycosylated enzymes (data not shown), suggesting that a loss of negative charges, possibly carried out by sialylated glycan chains [56], was associated with the loss in mass. 

Compared with native EeAChE, the specific activity of deglycosylated EeAChE was found to be virtually unaltered (data not shown), but inhibition of deglycosylated EeAChE by Fab403 led to near one order of magnitude *lower* IC50 value, with no change in the residual activity recorded at high Fab concentration (Figure 2C). The same observation was made with Fab409, prepared from IgG409 that was initially described as “similar in [ ] many respects” to IgG403 (data not shown) [32]. The gain in affinity suggests that one or several N-glycan chains proximal to the Fab403 (or Fab409) binding site in the PAS region hinder(s) proper recognition of the original epitope, although all three Fabs were generated against naturally glycosylated EeAChE [32]. In contrast, inhibition of the deglycosylated enzyme by Fab408 and Fab410 showed no difference in the IC50 and residual activity values. This is consistent with only partially overlapping binding loci for Fab403 and Fab410 at the PAS, respectively close to and remote from the hindering N-glycan(s), and with a fully distinctive binding site for Fab408 in the BDR.

TcAChE and human, bovine and rat AChEs, which share *ca.* 60% sequence identity with EeAChE and bear three to four N-glycosylation sites, are insensitive to the Elec mAbs [32,33]. Butyrylcholinesterase from human plasma (HuBChE) shares *ca.* 50% sequence identity with EeAChE and bears nine N-glycosylation sites [57,58], of which two, Asn241 and Asn341, are respectively located in the PAS region and the BDR. In fact, no Fab binding to either native or deglycosylated HuBChE was observed using a native-PAGE mobility shift assay (data not shown), consistent with the sequence differences in these regions in the two enzymes.

### Crystal structure of Fab408 at 1.9 Å resolution and implications for binding the BDR of EeAChE

**Figure 3:**
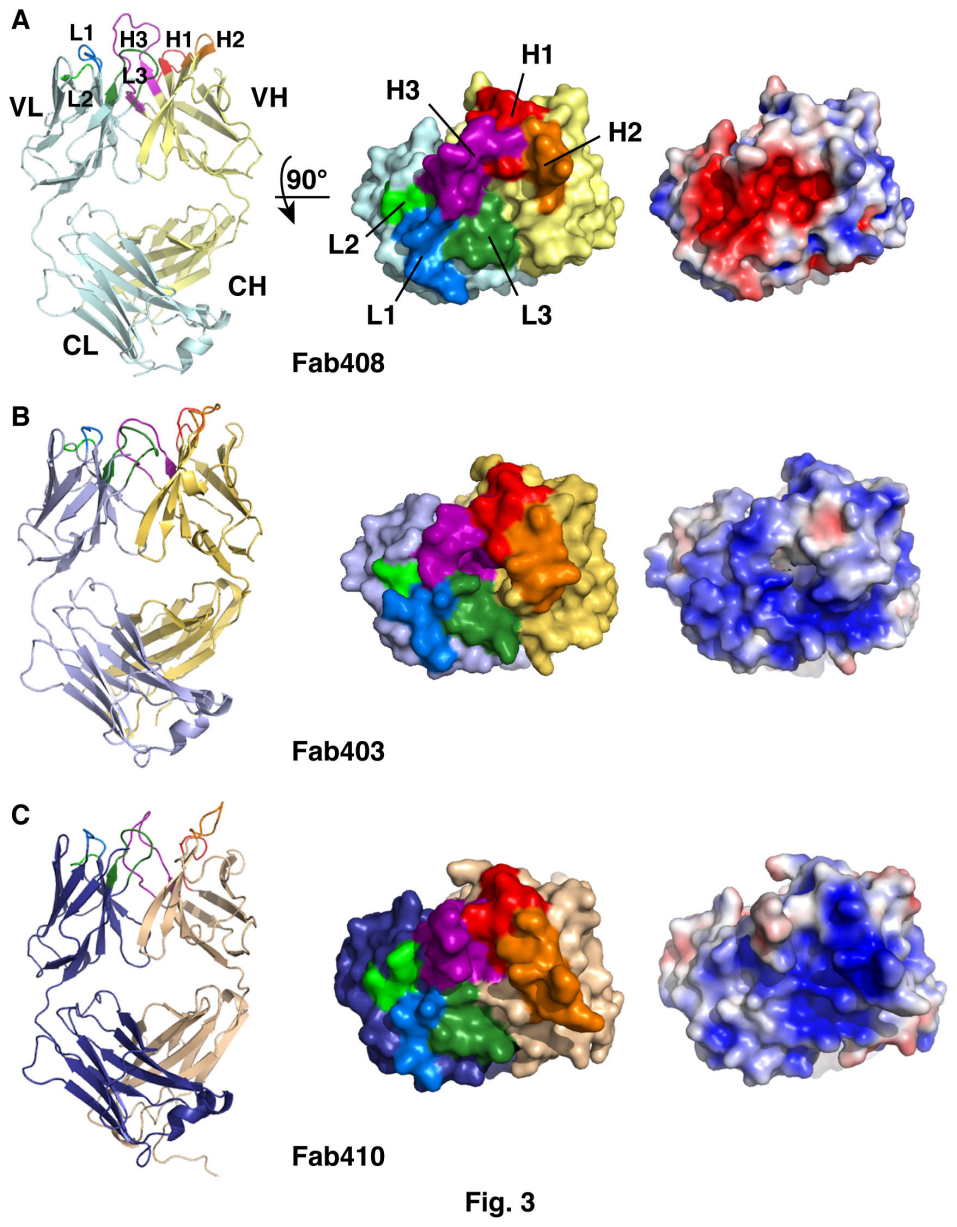
Structure of Fab408 and homology models of Fab403 and Fab410. **Left**: Overall views of (**A**) Fab408, with a light blue L chain and yellow H chain; (**B**) Fab403, with a violet L chain and orange H chain; (**C**) Fab410, with a dark blue L chain and salmon H chain, displayed with their N-terminal variable region on top and C-terminal constant region at bottom. CDR-L1, CDR-L2, CDR-L3 are displayed in blue, light green, dark green and CDR-H1, CDR-H2, CDR-H3 in red, orange, purple. The extended CDR-H3 in Fab408 is clearly visible. **Center**: Close-up views of the combining sites, displayed as molecular surfaces with the CDRs colored as on the left panels and labeled. Differences in the sizes of the CDRs and the shapes of the combining surfaces (pocket in Fab408 *versus* extended surfaces in Fab403 and Fab410) are evident. **Right**: Distribution of the electrostatic potentials mapped on the Fab molecular surfaces at -3 kT/e (red) to +3 kT/e (blue) (same orientation as on the central panels). Note the electronegative combining site in Fab408 *versus* the electropositive combining sites in Fab403 (centered around the L-chain CDRs) and Fab410 (centered around the H-chain CDRs).

The structure of Fab408, the first known peptidic ligand targeting the BDR of an AChE, shows the canonical β-sandwich Ig fold and an elbow angle of 151.6° between the pseudo-dyad axes relating the variable (VH and VL) and constant (CH and CL) domains of the two chains (Figure 3). The combining site is formed by the six CDRs emerging from the Fab variable domains (Figure 1 & Figure 3A). In the VL domain, the side chains of CDR-L1 residues Asn28, Asp30 and Tyr32 and of all CDR-L2 and CDR-L3 residues are oriented towards the solvent, with CDR-L3 being stabilized by two ionic interactions involving the His90 imidazole ring, the Thr93 carbonyl and the Thr97 hydroxyl. In the VH domain, Ser28 provides the only solvent-exposed side chain in CDR-H1 while the neighboring Tyr27 and Phe29 are totally buried within the molecule. The tip of CDR-H2 is bent inward. CDR-H3 consists of a long, 17-residue loop largely exposed to the solvent, except for residues Tyr110 and Phe111 at its base that contribute to the tight assembly of the H and L chains.

In Fab408, all six CDRs display local pI values below 7.0 (Figure S1B in File S1). As a result, the ~1000 Å^2^ surface defined by the CDRs is mostly populated by *anionic* side chains (Figure 3B). This *electronegative* patch confers on the Fab408 molecule a clear anisotropic distribution of surface charges with a moderate dipole moment of 484 Debyes and a vector oriented toward the H-chain N-terminus, *opposite to the CDRs*. This is consistent with the Fab408 neutral pI value (Figure 1; Table 1) and its interaction with the BDR of EeAChE, which is remote by more than 100° from the axis linking PAS residue Trp281 (homologous to mAChE Trp286) to active site residue Ser202 (mAChE Ser203) and roughly aligns with the dipole vector of the subunit (cf. below).

At the center of the Fab408 combining site, the interface between the VH and VL domains forms a small pocket of 10-12 Å diameter lined by side chains from the protruding CDR-H2 and CDR-H3 and the shorter CDR-L3 (Figure 3CD). Solvent-exposed CDR-H2 residues Ser54, Thr55, Arg57, CDR-H3 residues Thr103, Leu105, Glu107 and the CDR-L3 Thr93-Thr94 pair are well positioned for interaction with the BDR surface. It has been reported that 70% of an antibody contacts with its antigen involve contributions of Tyr>Asp>Asn>Ser>Glu>Trp side chains, while 15% of the antigen contacts with the antibody are triggered by Arg side chains [59,60]. On one side of the Fab408 pocket, the long and flexible CDR-H3, which encompasses a Glu-Asp-Trp-Tyr tetrapeptide (i.e. four of the six signature residues above defined) and a net negative charge, appears as a suitable candidate for interaction with the BDR of EeAChE. Yet, only cooperative binding of two (or more) CDRs at the BDR, perhaps with accommodation of a protruding surface motif within the Fab central pocket, would fulfill the requirement for an average antibody-antigen interfacial area of ~1700 Å^2^ [60]. In turn, the combined contribution of aromatic and charged side chains appears consistent with the subnanomolar IC50 of the complex (Table 1). A likely scenario would involve primary contribution of a few major anchoring interactions along with complementary contribution of several minor stabilizing interactions [61].

Mutagenesis studies performed using chimeras of EeAChE and rat AChE, which is not inhibited by the Elec mAbs, along with site-specific substitutions, pointed to the EeAChE 15-residue peptide Glu484-Arg498, which corresponds to the mAChE Asp460-Gln474 and TcAChE Val453-Arg467 peptides in the α(1)8,9-α(2)8,9 surface loop (Figure S2 in File S1), as a binding site for Elec408 [33]. In this peptide, the Leu487-Thr490 segment, which contains a consensus sequence for N-glycosylation at Asn488, and the neighboring Glu493 residue are largely conserved amongst AChE species and in HuBChE, while the other 11 residues are more variable. Moreover, Arg486 is unique to EeAChE amongst all AChE species (although it is found in HuBChE) (Figure S2 in File S1), while replacing either Leu491 or Glu494 by their respective Val and Arg counterpart in rat AChE abolished EeAChE inhibition by Elec408 [33]. However, should EeAChE residue Asn488 be glycosylated, as are the corresponding residues in recombinant mAChE and HuBChE and in natural TcAChE [15,58,62], it would have to be located out of the Fab408 binding site, since PNGase treatment of EeAChE does not alter the Fab408 binding and inhibition properties (cf. above).

In fact, at the tip of the long and flexible CDR-H3 of Fab408, Glu107 is well positioned to form a salt bridge with the Arg486 guanidinium. Using this possible interaction as a pivotal anchor, and keeping Asn488 away, a search for complementary interactions between Fab408 and EeAChE pointed to the polar Ser54-Thr55 residue pair at the tip of CDR-H2, positioned close to CDR-H3 in the combining site, while small side chains protruding from CDR-L3, opposite to CDR-H3 across the central pocket, are suitably positioned to confer additional polar interactions with Arg494 (Figure 3D). However, considering the limited amount of experimental constraints available (in contrast to the situation for Fab403, cf. below), modeling of a Fab408-EeAChE complex was not attempted. 

### Model of Fab403 and implications for binding the PAS of EeAChE

In the model of Fab403, the side chains of several residues in each of the six CDRs point towards the solvent (cf. Experimental Procedures). Analysis of the Fab403 CDR sequences reveals a distinctive repartition of theoretical pI values: for CDR-H1, CDR-H3 and CDR-L2 these values are in the 5.2-6.0 range while for CDR-H2, CDR-L1 and CDR-L3 they are in the 8.0-8.5 range (Figure 1; Figure S1B in File S1). As a result, the ~1600 Å^2^ combining site of Fab403 is mostly populated by *cationic* side chains (Figure 3B). This *electropositive* patch, combined with an uneven repartition of negatively charged residues within the rest of the molecule, confers on the Fab403 molecule a clear anisotropic distribution of surface charges, with a marked dipole moment of 1270 Debyes (i.e., ~2.6-fold higher than that of Fab408) and a dipole vector roughly aligned along L-chain strand β4 and oriented *toward the L-chain CDRs* (Figure 3B). This observation is consistent with the observed high pI value of Fab403 (Figure 2; Table 1) and its interaction with the PAS of EeAChE [32], as does the cationic Fas2 molecule, with its dipole vector oriented towards the tips of the positively charged loops I and II [26,63]. The three cationic CDRs in Fab403 also encompass residues suitable for promoting hydrophobic interaction, as found for loops I and II of Fas2. However, the conformation of CDR-H2 does not reflect its sequence homology with Fas2 loop I (cf. above), since a two residue shift along the backbone places residues His54-Thr55, instead of Thr56-Thr57, at the tip of the CDR-H2 loop to mimic Fas2 loop I residues Thr8-Thr9 (Figure S3 in File S1). As a result, compared with the Arg9 side chain that protrudes at the edge of Fas2 loop I, the Arg59 side chain lies ~20 Å away at the base of CDR-H2 in a position likely to promote distinctive interactions with the AChE PAS.

The conserved electrostatic properties of Fab403 and Fas2 correlate with functional similarities. Indeed, Elec/Fab403 and Fas2 bind the PAS competitively with each other and with several other PAS ligands, and both the Elec/Fab403- and Fas2-EeAChE complexes display low residual activities (Table 1) [32]. These features denote comparable (but not identical, as suggested by the slightly different residual activities) capacities of the two peptidic ligands to cover a large surface area in the PAS region, occlude the active site gorge entrance, and prevent substrate access to the active center, despite the 100-fold difference in their respective Kd values. Analysis of rat/EeAChE chimeras designed to map the binding site of Fab403 at the EeAChE surface pointed to residue Ser75 (mAChE Leu76, TcAChE Gln74) in the long Ω loop (loop b3-β3) on one side of the gorge entrance, and to residues Gln279 (mAChE His284, TcAChE Val277) and Leu282 (mAChE His287, TcAChE Asn280) in the facing short Ω loop (loop α3(6,7)-α4(6,7)) on the other side of the gorge entrance, as most critical binding partners [33]. The corresponding mAChE or TcAChE residues are involved in binding Fas2, propidium, decamethonium, BW284C51, and ATCh [7,8,13,20,21,26]. Finally, deglycosylation of EeAChE enhanced the Fab403 affinity by almost one order of magnitude (Figure 2D; Table 1), suggesting that one or several N-glycan chains are proximal to the combining site. Of the six consensus sites for N-glycosylation in EeAChE, one, at Asn345 (mAChE Asn350, TcAChE Ser343), is located in the PAS region. All together, these features suggest that the formation and conformation of the Fab403-EeAChE complex obey electrostatics, chemical and steric constraints close to those dictating the formation and conformation of the Fas2-AChE complex (cf. Introduction), and represent suitable constraints to frame and validate a flexible docking study (cf. Experimental Procedures, and below).

**Figure 4:**
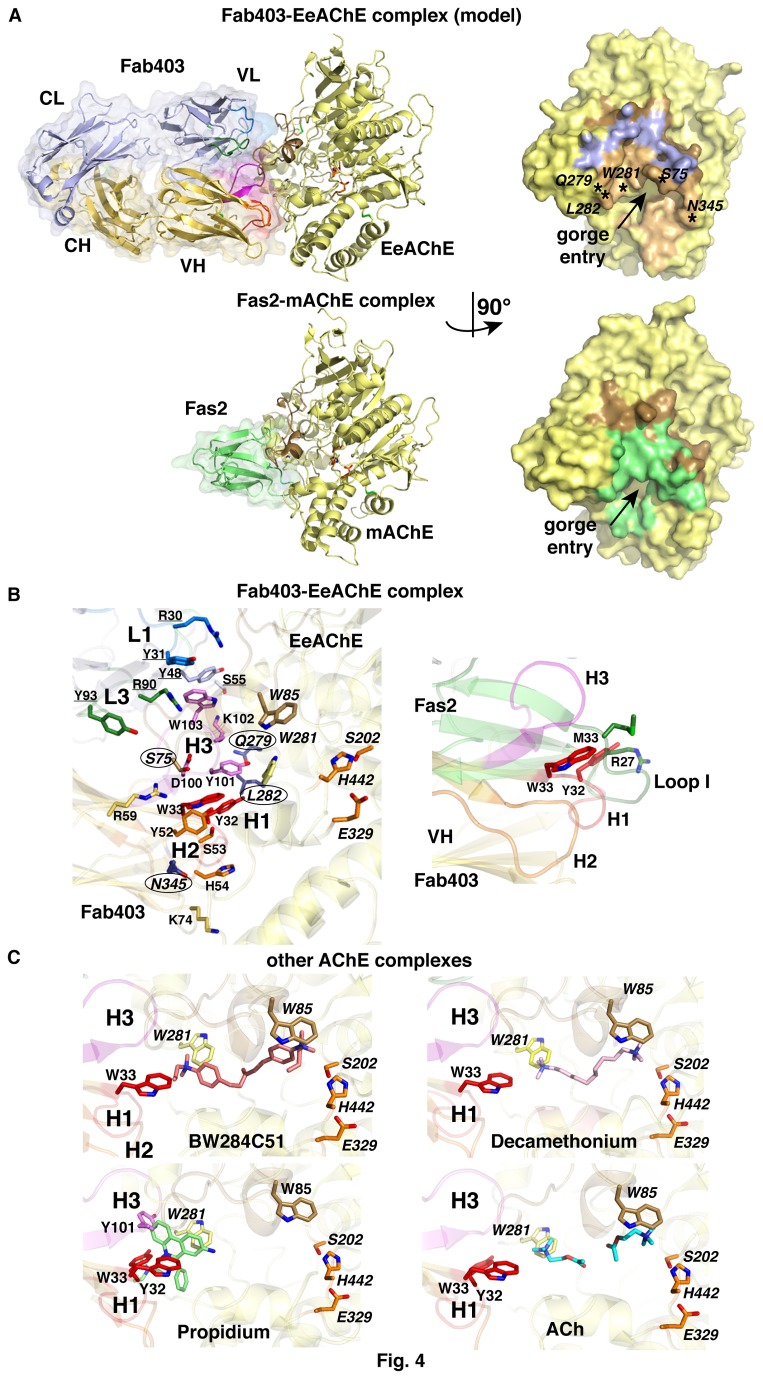
Docking model of the Fab403-EeAChE complex and structural comparisons. (**A**) Overall views of (top left) the most representative docking model of the Fab403-EeAChE complex and (top right) the buried interface at the EeAChE surface, oriented 90° from each other. The EeAChE subunit is displayed in yellow and the L and H chains of Fab403 in violet and orange, respectively. The AChE catalytic residues are displayed in orange at the center of the subunit and the long Ω loop in brown at the subunit surface / complex interface. The positions for Trp281 (Trp279/286 in TcAChE/mAChE), for Ser75, Gln279, and Leu282 whose substitution prevents Fab403 binding, and for Asn345 whose deglycosylation enhances Fab403 binding, are indicated (stars) and labeled. The arrow points to the entry of the active site gorge. For comparison, shown are the overall views of (bottom left) the crystal structure of the Fas2-mAChE complex (accession code, 1KU6) and (bottom right) the buried interface at the mAChE surface, oriented 90° from each other. The mAChE subunit is displayed in yellow and Fas2 in green. On the right panels, the AChE surfaces buried by the bound Fab403 L chain (590 Å^2^) and H chain (1000 Å^2^) and by Fas2 (1029 Å^2^) are colored accordingly to the ligands. Bound Fab403, but not bound Fas2, almost completely covers the long Ω loop. The similar surface areas buried by the Fab H chain and by Fas2 at the AChE PAS denote similar plugging of the gorge entry. (**B**) Close-up view (left) of the Fab403-EeAChE complex interface viewed as in (a) and showing the key EeAChE and main Fab interacting side chains, and close-up overlay (right) of Fab403 and Fas2 as bound to AChE, showing the distinct lengths of Fas2 loop I and Fab403 CDR-H1 and distinct positioning of their side chains at the gorge entrance. Standard and underlined labels are for residues in the Fab403 H and L chains, respectively, and italicized labels for EeAChE residues with encircled Ser75, Leu282, Gln279, and Asn345. (**c**) Close-up views of the Fab403-EeAChE complex overlaid with BW284C51 (salmon inhibitor molecule; 1E3Q) and decamethonium (pink; 1ACL) as bound to TcAChE, and with propidium (green; 1N5R) and ACh (blue; 2HA4) as bound to mAChE and its Ser203Ala mutant, respectively. PAS residue Trp281 is displayed in yellow, oxyanion hole residue Trp85 in brown, and the catalytic triad residues in orange. Steric clashes between residues in the Fab403 CDR-H1 (red) and CDR-H3 (magenta) and each of the four organic PAS ligands are evident.

The only EeAChE form available in suitable amounts and molecular homogeneity for structural studies is the covalent tetramer enzymatically processed from the natural asymmetric (A12) form (cf. Reference [S5] in File S1), whose high flexibility hinders generation of well diffracting crystals and hence, high-resolution description of an EeAChE subunit [55]. Therefore, based on the high sequence conservation amongst AChE species, we generated a homology model of the subunit. Analysis of the electrostatic potentials at the molecular surface of the EeAChE subunit evidenced a marked anisotropy of charge distribution, associated with a dipole vector roughly aligned with the gorge path and oriented opposite to the PAS, as observed for other AChE species [26,63-65] (data not shown). The permanent dipole moment, of 1555 Debyes, is 1.8-fold and 0.9-fold greater than those of mAChE and TcAChE, of 883 and 1760 Debyes, respectively. Software-assisted docking of the modeled Fab403 onto the modeled EeAChE subunit led to seven ensembles of four energetically best models of a complex, of which one was predominantly found in the lowest HADDOCK scores. In this model, the dipole vectors of the two partners are roughly aligned and the Fab403 H and L chains contribute 640 Å^2^ and 510 Å^2^, respectively, to the buried interfacial surface area. The combining site of Fab403 traps the long Ω loop in EeAChE encompassing Ser75, with residues Tyr101-Trp103 in the very hydrophobic CDR-H3 being anchored central to the binding interface (Figure 4). In contrast to Fas2 residues Arg27 and Met33 at the tip of loop II, which occlude the entrance of the active center gorge [13,26], Fab403 residues Tyr101 and Lys102 in the shorter CDR-H3 are shifted slightly outward so that gorge occlusion in the Fab403 complex is not as tight as in the Fas2 complex, as reflected in the slightly different residual activities (cf. Introduction) (Figure 4B). Vicinal to these anchoring residues, CDR-H1 residues Ser31-Trp33 and CDR-H2 residues Tyr52 and His54 contribute to the binding interface through interactions with EeAChE residues flanking the PAS. At the periphery of the binding interface, CDR-L1 residues Arg30 and Tyr31 and CDR-L2 residues Asn52 and Leu53 provide additional contact points through polar and non polar interactions with EeAChE residues Gln279 and Leu282, while CDR-L3 only moderately contributes to the binding interface. Non-CDR residues Tyr48, Ser55 and Ala59 in the L chain also significantly contribute to the binding interface. Finally, close proximity of CDR-H2 with PAS residue Asn345 is largely consistent with the greater affinity of Fab403 for deglycosyled EeAChE, compared with native EeAChE.

To validate this docking procedure, we also docked the Fas2 molecule onto the mAChE structure and the EeAChE model using a limited number of ambiguous interaction restraints (see Experimental procedures). Two and three ensembles of models were respectively obtained, from which the first and second best solutions generated by HADDOCK matched the reference Fas2-mAChE complex structure, with rmsd values in the 0.6-1.1 Å range (data not shown). Relevance of the modeled Fab403-EeAChE complex was further assessed by comparison with the crystal structures of mAChE or TcAChE complexes with the organic PAS ligands propidium [15] and acetylcholine [7,8] and with the bifunctional ligands BW284C51 [21] and decamethonium [20], which bind EeAChE competitively with Elec403 [32] (Figure 4C). In fact, steric clashes were observed between each of the four bound ligands and CDR-H3 residue Tyr101 and, occasionally, neighboring Lys102 in bound Fab403, thereby consistent with the competition data. Finally, the Tyr71Met mutation in the long Ω loop of BfAChE (Figure S2 in File S1) associated with subtle substitutions in the short Ω loop at positions that contribute to the binding interface in the model, e.g. Leu282Ser and Ser287Lys, are likely to contribute to absence of BfAChE inhibition by Fab403 [32].

### Model of Fab410 and implications for binding the PAS of EeAChE and BfAChE

In the model of Fab410, the side chains of several residues in each of the six CDRs point towards the solvent (cf. Experimental Procedures). As observed for Fab403 (cf. above), analysis of the Fab410 CDRs reveals a distinctive repartition of theoretical pI values: for CDR-H1, CDR- H3 and CDR-L1 these values are in the 4.0-5.2 range, while for CDR-H2 and CDR-L2 they are in the 8.7-11.0 range; only CDR-L3 has a theoretical pI value close to neutrality (Figure 1; Figure S1B in File S1). As a result, the ~1000 Å^2^ combining site of Fab410 is markedly *electropositive*, and its dipole moment, of 892 Debyes (i.e., 1.8 greater and 1.4-fold smaller than those of Fab408 and Fab403, respectively), is associated with a vector roughly aligned along H-chain strand β4 and oriented *toward the H-chain CDRs*, instead of the L-chain CDRs as found in Fab403 (Figure 3C). This observation is consistent with the observed high pI value of Fab410 (Table 1) and its binding at the EeAChE PAS competitively with Fab403 and Fas2 binding [32]. In fact, the highly electropositive CDR-H2 appears as a prime candidate to provide the attractive energy and primary anchor point for Fab410 at the rim of the gorge, while the protruding CDR-H3, with the Tyr104-Tyr105-Asp106 residue triplet at its tip, is suitably positioned to provide additional contact points at the periphery of the PAS, thereby extending the interfacial surface area and number of stabilizing interactions for high affinity binding. 

Experimental information that can help delineate the Fab410 binding site at the EeAChE surface points to i) absence of binding competition between Fab410 and the smaller, organic PAS ligands, ii) a residual activity of the Fab410-EeAChE complex slightly higher than that of the Fab403-EeAChE complex, iii) critical contribution of Ser75, but not Gln279 and Leu282, to Fab410 binding (Figure S2 in File S1; cf. Figure 4A), which together suggested that Fab410 binds only one side of the gorge rim, opposite to Trp281 (mAChE Trp286, TcAChE Trp279), and that none of its CDRs occludes the gorge entrance [32,33]. Moreover, unaltered binding of Fab410 upon deglycosylation of EeAChE, denotes Asn345 positioning out of the Fab410 binding site (Figure 2; cf. Figure 4A). These constraints preclude Fab410 binding to three quarters of the EeAChE PAS region, yet they do not provide sufficient information as to the binding position and orientation of Fab410 at the periphery of the gorge entrance. Therefore, modeling of the Fab410-EeAChE complex was not attempted.

In contrast to Elec-403, Elec-410 inhibits BfAChE with a lower affinity compared to EeAChE [32,34]. Apart from unpredictable structural variations between the two AChE species, this feature denotes differences in the interaction networks at the two PAS regions. Comparison of the EeAChE and BfAChE sequences in the PAS surroundings points to Tyr71Met, Ser80Gln and Met90Gly substitutions, close to the conserved Ser75 and on the solvent-exposed face of the long Ω loop, and to a Ser287Lys substitution in the short Ω loop, as likely candidates for dictating a less favorable complex interface (Figure S2 in File S1). Moreover, of the five consensus sites for N-glycosylation of BfAChE, three, at Asn343 (corresponding to EeAChE Asn345), Asn453 (EeAChE Glu484) and Asn457 (EeAChE Asn488), are located on each side of the gorge entrance (Figure S1 in File S1). Presence of glycan chains at all three positions, compared to only two on EeAChE, would provide additional constraints onto Fab410 positioning in the PAS region of BfAChE.

*In conclusion*, comparative analysis of a crystal structure of Fab408, which inhibits EeAChE by binding to the BDR, and of models of its Fab403 and Fab410 congeners, which inhibit EeAChE by binding the PAS, points to distinctive surface topographies and anisotropic repartitions of charges of their combining sites, consistent with their respective target sites at the EeAChE surface and the distinctive residual activities of their EeAChE complexes. A flexible docking model of the Fab403-AChE complex, based on the available functional data and validated through independent modeling of Fas2-AChE complexes, pictures a likely mode of Fab403 binding, reminiscent of that of the Fas2 toxin. This comprehensive study documents the molecular peculiarities of Fab403 and Fab410, as the largest peptidic ligands directed towards the PAS, and of Fab408, as the first peptidic ligand directed toward the BDR. The structure of Fab408 also provides a unique template for the design of new, specific modulators of AChE, to be assayed as therapeutic agents to treat neuromuscular, ophthalmic, or cognitive disorders associated with cholinergic deficiencies [66]. Further structural analysis of these “Elec” mAbs bound to AChE should provide detailed picturing of the individual molecular determinants at the AChE surface and mechanisms involved into regulation of catalysis. 

## Supporting Information

File S1 **Supplemental Experimental Procedures and References [S1-S17], Table S1, Figure S1 (panels A1-3 and B), Figure S2 and Figure S3.**
(PDF)
