# Supplementary material for: Molecular Characterization of Monoclonal Antibodies that Inhibit Acetylcholinesterase by Targeting the Peripheral Site and Backdoor Region
Source: PLoS One. 2013 Oct 11;8(10):e77226. doi: 10.1371/journal.pone.0077226 (PMC3795623; doi:10.1371/journal.pone.0077226)
Supplement: File S1 — Supplemental Experimental Procedures and References [S1-S17], Table S1, Figure S1 (panels A1-3 and B), Figure S2 and Figure S3. (PDF) [file pone.0077226.s001.pdf]

***Supporting Information to***

# Molecular characterization of monoclonal antibodies that inhibit acetylcholinesterase by targeting the peripheral-site and backdoor regions

**Yves BOURNE[1£], Ludovic RENAULT[2£], Sosthène ESSONO[3], Grégoire MONDIELLI[4], Patricia LAMOURETTE[3], Didier BOQUET[5], Jacques GRASSI[3], & Pascale MARCHOT[1,2,4]***

[1]Architecture et Fonction des Macromolécules Biologiques (AFMB), CNRS/Aix-Marseille Université, Campus Luminy, Marseille, France. [2]Ingénierie des Protéines, CNRS/Aix-Marseille Université, Faculté de Médecine - Secteur Nord, Marseille, France. [3]CEA, iBiTecS, Service de Pharmacologie et Immunologie (SPI), Laboratoire d'Etude et de Recherche en Immunoanalyse (LERI), Gif-sur-Yvette, France. [4]Centre de Recherche en Neurobiologie-Neurophysiologie de Marseille (CRN2M), CNRS/Aix-Marseille Université, Faculté de Médecine - Secteur Nord, Marseille, France. [5]CEA, iBiTecS, Service de Pharmacologie et Immunologie (SPI), Laboratoire d'Ingénierie des Anticorps pour la Santé (LIAS), Gif-sur-Yvette, France.

[£]Equal contributors to this work.

*Corresponding author: pascale.marchot@univ-amu.fr

---

## Supplemental Experimental Procedures

*Materials.*

The prepacked Superdex-200 HR-10/30 and HL-26/60 columns and protein-G and protein-A Sepharose HiTrap units (1 ml) and the calibration markers for gel filtration were from GE Healthcare. PEG-6000 was from Hampton Research and Na cacodylate from Fluka. The molecular weight standards for SDS-PAGE and all others biochemical reagents were from Sigma-Aldrich.

*Protein purification and preparation.*

Elec403, Elec408 and Elec410 were purified from the ascitic fluids in a single step of affinity FPLC on HiTrap protein-G (GE Healthcare) equilibrated with 20 mM NaP, pH 7.0, and eluted with 100 mM glycine, pH 2.7, with immediate neutralization of the eluant with 1 M Tris, pH 9.0 (55 µL/ml). The purified IgGs were dialyzed against 20 mM NaP, pH 7.0, and concentrated by ultrafiltration.

The Fabs were obtained by papaine cleavage of the purified IgGs using papain (25.8 IU/mg) from Sigma-Aldrich, a papain-to-IgG ratio of 1:25 (w/w), and 1 mM EDTA and 1 mM β-mercaptoethanol (12-20 h, 37°C); the reaction was stopped with iodoacetamide 6 mM. The cleavage reactants and products were separated by gel-filtration FPLC on prepacked Superdex-200 (GE Healthcare) equilibrated and eluted with 0.02 M NaP, pH 7.2. The coeluting Fab and Fc fragments were separated through several steps of affinity FPLC on HiTrap protein-A (GE Healthcare) equilibrated in the same buffer, with recovery of the non-retained Fab in the flow through and expulsion of the retained Fc using 100 mM citric acid, pH 5.0. Homogeneity of the purified Fab was assessed by SDS- and native-PAGE and by MALDI-TOF mass spectrometry (cf. below). The Fabs were dialyzed against 50 mM Tris pH 7.5, 50 mM NaCl, 0.01% (w/v) $NaN_3$, and concentrated by ultrafiltration.

EeAChE, as a mixture of soluble asymmetric forms, was isolated from homogenized electric organs by affinity chromatography and subjected to controlled tryptic cleavage to release the constitutive covalent tetramers [S5]. The tetramers were purified from the tryptic mixture by gel filtration in 100 mM NaP, pH 7.4, 400 mM NaCl, 0.01% (w/v) $NaN_3$ [55]. Homogeneity was assessed by SDS- and native-PAGE (cf. below). The enzyme was dialyzed against 50 mM NaP pH 7.4, 50 mM NaCl, 0.01 % (w/v) $NaN_3$ (buffer A) and concentrated by ultrafiltration; it was stored on ice. Native and deglycosylated HuBChE samples were gifts from Dr. Ashima Saxena (WRAIR, Silver Spring, MD).

*Biochemical and functional analyses.*

SDS- and native-PAGE used a PhastSystem apparatus (GE Healthcare), homogenous 12.5% and 7.5% gels, respectively, migration towards the anode and Coomassie blue staining. The SDS-PAGE samples were boiled for 5 min in the presence of 2.5% (w/v) SDS with (reducing conditions) or without (non-reducing conditions) 5% (v/v) β-mercaptoethanol. Native-PAGE mobility shift assays used Fab-EeAChE or Fab-HuBChE complexes formed in solution at a ~1:1 molar ratio (3 h incubation, room temperature). Isoelectric focusing used the same apparatus and pI 3-9 gels.

MALDI-TOF MS was performed on a Voyager-DE™RP BioSpectrometer Workstation (Perseptive Biosystems) in the positive linear mode using ~10 pmol/0.5 µl samples mixed with, as a matrix, 0.5 µl of sinapinic acid at 10 mg/ml in TFA/acetonitrile/water 0.1:0.6:0.3 (v/v/v), and the dried-droplet method. The samples were desorbed with a 337 nm nitrogen laser.

AChE activities were recorded for 5 min in duplicate or triplicate on a UNICAM 8700 spectrophotometer (Thermo Optek) using 10 pM EeAChE, 1.25 mM acetylthiocholine iodide (~10 x Km) and 0.33 mM dithiobis(2-nitro-benzoic acid) in 100 mM sodium phosphate, pH 8.0, 0.1mg/ml BSA (λ = 412 nm) [S16]. The Fab/AChE mixtures were

incubated overnight (equilibrium analysis) or for selected time intervals (kinetics analysis) under mild agitation at room temperature before recording of fractional activity. Data analysis used GraphPad Prism 4.0.

*N-linked carbohydrate removal.*

Deglycosylation of the EeAChE tetramer (~3 mg/ml) in native conditions was performed in buffer A using PNGaseF (1,800,000 $U.mg^{-1}$) from BioLabs, a PNGaseF-to-tetramer ratio of 1:200 (w/w) and overnight incubation at 25°C. A control sample for protein integrity was incubated in the absence of PNGaseF. A control sample for total deglycosylation was prepared on denatured and reduced EeAChE: briefly, the EeAChE tetramer (~20 µg in 6.25 µl buffer A) was boiled for 10 min in the presence of 40 mM DTT and 0.5% (w/v) SDS, cooled down, added with 1% (v/v) Nonidet P-40, then incubated in the presence of PNGaseF (~2 µg, 3600 U) for 1 h at 37°C. The native and reduced deglycosylated samples were analyzed comparatively with the unaltered native enzyme by SDS-PAGE in reducing conditions and native-PAGE.

*Crystallization of Fab408 and data collection.*

Crystallization was achieved at 20°C by vapor diffusion using Fab408 at 10 mg/ml, 1 µl hanging drops and a protein-to-well solution ratio of 1:1. Plate crystals grew spontaneously within 2 days with 18% (v/v) PEG-6000 in 100 mM Na cacodylate, pH 6.0, as the well solution. Crystals were flash-cooled in the nitrogen gas stream after successive short soaks in the well solution supplemented with 7.5%, 15% and 25% (v/v) glycerol, and were stored in liquid nitrogen. Diffraction data were collected at 100 K at the ESRF (Grenoble, France), processed with XDS [S17], and scaled and merged with SCALA. Despite the numerous attempts, no suitable crystals were obtained from Fab403 and Fab410.

**Supplemental references to the Introduction**

S1. Fambrough DM, Engel, AG, Rosenberry TL (1982) Acetylcholinesterase of human erythrocytes and neuromuscular junctions: homologies revealed by monoclonal antibodies. Proc Natl Acad Sci U S A 79: 1078-1082.

S2. Brimijoin S, Mintz KP, Prendergast FG (1985) An inhibitory monoclonal antibody to rabbit brain acetylcholinesterase. Studies on interaction with the enzyme. Mol Pharmacol 28: 539-545.

S3. Mintz KP, Brimijoin S (1985) Monoclonal antibodies to rabbit brain acetylcholinesterase: selective enzyme inhibition, differential affinity for enzyme forms, and cross-reactivity with other mammalian cholinesterases. J Neurochem 45: 284-292.

S4. Sorensen K, Brodbeck U, Rasmussen AG, Norgaard-Pedersen B (1987) An inhibitory monoclonal antibody to human acetylcholinesterases. Biochim Biophys Acta 912: 56-62.

S5. Grassi J, Frobert Y, Lamourette P, Lagoutte B (1988) Screening of monoclonal antibodies using antigens labeled with acetylcholinesterase: application to the peripheral proteins of photosystem 1. Anal Biochem 168: 436-450.

S6. Wolfe AD (1989) The monoclonal antibody AE-2 modulates fetal bovine serum acetylcholinesterase substrate hydrolysis. Biochim Biophys Acta 997: 232-235.

S7. Ashani Y, Gentry MK, Doctor BP (1990) Differences in conformational stability between native and phosphorylated acetylcholinesterase as evidenced by a monoclonal antibody. Biochemistry 29: 2456-2463.

S8. Olson CE, Chhajlani V, August JT, Schmell ED (1990) Novel allosteric sites on human erythrocyte acetylcholinesterase identified by two monoclonal antibodies. Arch Biochem Biophys 277: 361-367.

S9. Gentry MK, Saxena A, Ashani Y, Doctor BP (1993) Immunochemical characterization of anti-acetylcholinesterase inhibitory monoclonal antibodies. Chem Biol Interact 87: 227-231.

S10. Wolfe AD, Chiang PK, Doctor BP, Fryar N, Rhee JP, et al. (1993) Monoclonal antibody AE-2 modulates carbamate and organophosphate inhibition of fetal bovine serum acetylcholinesterase. Mol Pharmacol 44: 1152-1157.

S11. Gentry MK, Moorad DR, Hur RS, Saxena A, Ashani Y, et al. (1995) Characterization of monoclonal antibodies that inhibit the catalytic activity of acetylcholinesterases. J Neurochem 64: 842-849.

S12. Saxena A, Hur R, Doctor BP (1998) Allosteric control of acetylcholinesterase activity by monoclonal antibodies. Biochemistry 37: 145-154.

S13. Sharma KV, Bigbee JW (1998) Acetylcholinesterase antibody treatment results in neurite detachment and reduced outgrowth from cultured neurons: further evidence for a cell adhesive role for neuronal acetylcholinesterase. J Neurosci Res 53: 454-464.

S14. George KM, Montgomery MA, Sandoval LE, Thompson CM (2002) Examination of cross-antigenicity of acetylcholinesterase and butyrylcholinesterase using anti-acetylcholinesterase antibodies. Toxicol Lett 126: 99-105.

S15. Guo CZ, Wu JH, Wang YX, Hu YD, Li S, et al. (2003) Molecular simulation of a single-chain antibody against AChE to explore molecular basis of inhibitory effect of 3F3 McAb on enzyme activity. Acta Pharmacol Sin 24: 460-466.

**Supplemental references to the Supplemental Results and Experimental Procedures**

S16. Ellman GL, Courtney KD, Andres V, Feather-Stone RM (1961) A new and rapid colorimetric determination of acetylcholinesterase activity. Biochem Pharmacol 7: 88-95.

S17. Kabsch W (1993) Automatic processing of rotation diffraction data from crystals of initially unknown symmetry and cell constants. J Appl Cryst 26: 795-800.

## Supplemental Table

**Table S1. Data collection and refinement statistics.**

| | Fab408 |
|---|---|
| **Data collection** [a] | |
| Space group | $C222_1$ |
| Cell parameters (Å) | a = 69.95, b = 129.11, c = 99.26 |
| Beamline (ESRF) | ID14-EH2 |
| Resolution range (Å) | 30.0 - 1.9 |
| Total observations | 211 893 |
| Unique reflections | 35 565 |
| Multiplicity | 6.0 (5.4) |
| Completeness (%) | 99.5 (97.3) |
| $<((I)/\sigma (I))>$ | 15.0 (3.8) |
| Rsym [b] | 6.6 (44.9) |
| B Wilson ($Å^2$) | 31.7 |
| **Refinement** [c] | |
| R-factor / R-free (%) | 20.8 (28.2) / 25.3 (37.2) |
| **R.m.s.d.** [d] | |
| Bonds (Å) / Angles (°) | 0.0097 / 1.34 |
| Chiral volume ($Å^3$) | 0.089 |
| **Mean B-factors (Å)** | |
| Main / Side chains | 41.8 / 43.8 |
| Solvent | 40.4 |
| **Ramachandran plot statistics** [e] | |
| % Residues in favored/outlier regions | 97.4/0.2 |
| PDB accession code | 2YMX |

[a] Values in parentheses are those for the highest resolution shell.
[b] Rsym = $\Sigma_{hkl}(\Sigma_i|I_{hkl}-\langle I_{hkl}\rangle|)/\Sigma_{hkl}|\langle I_{hkl}\rangle|$.
[c] R-factor = $\Sigma_{hkl}|\,|Fo|-|Fc|\,|/\Sigma_{hkl}|Fo|$. Rfree is calculated for 2% of randomly selected reflections excluded from refinement.
[d] Root-mean-square deviations from ideal geometry.
[e] Ramachandran plot statistics have been calculated with the MolProbity server

## Supplemental Figures

### VL 403

```
                              <--------------------------------------------- FR1 - IMGT ------------------------------
                              1              5                   10                  15                  20
                               E   I   F   L   T   Q   S   P   A   I   I   A   A   S   P   G   E   K   V   T   I   T   C
VL403                         gaa att ttt ctc acc cag tct cca gca atc ata gct gca tct cct ggg gag aag gtc acc atc acc tg
                                       L
AJ231224 Musmus IGKV4-90*01 F --- --- --g --- --- --- --- --- --- --- --- --- --- --- --- --- --- --- --- --- --- --- --

                              ------------> _________________ CDR1 - IMGT ______________ <---------------------------
                                    25                  30                  35                  40                  45
                                 S   A   S   S   S   V                               R   Y   M   H   W   Y   Q   Q   K
VL403                         c agt gcc agc tca agt gta ... ... ... ... ... ... ... cgt tac atg cac tgg tac cag cag aaa
                                                                                     S           N
AJ231224 Musmus IGKV4-90*01 F - --- --- --- --- --- --- ... ... ... ... ... ... ... a-- --- --- a-- --- --- --- --- ---

                               FR2 - IMGT  -------------------------> ______________ CDR2 - IMGT __________ <---------
                                                 50                  55                  60                  65
                               P   G   S   S   P   K   I   W   I   Y   G   I                             S   N   L   A
VL403                         cca gga tcc tcc ccc aaa ata tgg att tat ggt ata ... ... ... ... ... ... ... tcc aac ctg gc
AJ231224 Musmus IGKV4-90*01 F --- --- --- --- --- --- --- --- --- --- --- --- ... ... ... ... ... ... ... --- --- --- --

                              ------------------------------------------------------------- FR3 - IMGT ---------------
                                    70                  75                  80                  85                  90
                                 S   G   V   P       A   R   F   S   G   S   G           S   G   T   S   F   S   F   T
VL403                         t tct gga gtt cct ... gct cgc ttc agt ggc agt ggg ... ... tct ggg aca tct ttc tct ttc aca
AJ231224 Musmus IGKV4-90*01 F - --- --- --- --- ... --- --- --- --- --- --- --- ... ... --- --- --- --- --- --- --- ---

                              -------------------------------------------------------> ______________ CDR3 - IMGT _____
                                                95                  100                 104
                               I   N   S   M   E   A   E   D   V   A   T   Y   Y   C   Q   Q   R   S   S   Y   P   P   L
VL403                         atc aac agc atg gag gct gaa gat gtg gcc act tat tac tgt cag caa agg agt agt tac cca ccc ct
AJ231224 Musmus IGKV4-90*01 F --- --- --- --- --- --- --- --- --t --- --- --- --- --- --- --- --- --- --- --- --- ---

                              _____
                                 T   F   G   A   G   T   K   L   E   L   K   R
VL403                         c acg ttc ggt gct ggg acc aag ctg gag ctg aaa cgg
AJ231224 Musmus IGKV4-90*01 F
```

### VH 403

```
                              <--------------------------------------------- FR1 - IMGT ------------------------------
                              1              5                   10                  15                  20
                               Q   V   Q   L   Q   Q   P   G   A       E   L   V   K   P   G   A   S   V   K   L   S   C
VH403                         cag gtc caa ctg cag cag cct ggg gct ... gag ttg gta aag cct ggg gct tca gtg aag ttg tcc tg
AJ073939 Musmus IGHV1-64*01 F --- --- --- --- --- --- --- --- --- ... --- c-- --- --- --- --- --- --- --- --- --- --- --

                              ------------> _________________ CDR1 - IMGT ______________ <---------------------------
                                    25                  30                  35                  40                  45
                                 K   A   S   G   Y   T   F                   T   S   Y   W   M   H   W   V   K   Q   R
VH403                         c aag gct tct ggc tac act ttc ... ... ... ... acc agc tac tgg atg cac tgg gtg aag cag agg
AJ073939 Musmus IGHV1-64*01 F - --- --- --- --- --- --- --- ... ... ... ... --- --- --- --- --- --- --- --- --- --- ---

                               FR2 - IMGT  -------------------------> ______________ CDR2 - IMGT __________ <---------
                                                 50                  55                  60                  65
                               P   G   Q   G   L   E   W   I   G   M   A   Y   S   H           T   T   T   S   R   S   N
VH403                         cct gga caa ggc ctt gag tgg att gga atg gct tat tct cat ... ... act act act agt aga tcc aa
                                                                       I   H   P   N           S   G   S   T   N   Y
AJ073939 Musmus IGHV1-64*01 F --- --- --- --- --- --- --- --- --- --- at- c-- c-- a-- ... ... -g- gg- -g- -c- -ac -a- --

                              ------------------------------------------------------------- FR3 - IMGT ---------------
                                    70                  75                  80                  85                  90
                                 E   K   F   K       T   K   A   T   L   T   V   D   K   S   S   S   T   A   Y   M   Q
VH403                         t gag aag ttc aag ... acc aag gcc aca ctg act gta gac aaa tct tcc agc aca gcc tac atg caa
                                                     S
AJ073939 Musmus IGHV1-64*01 F - --- --- --- --- ... -g- --- --- --- --- --- --- --- --- --c --- --- --- --- --- --- ---

                              -------------------------------------------------------> ______________ CDR3 - IMGT ____
                                                95                  100                 104
                               L   S   S   L   T   S   E   D   S   A   V   Y   F   C   A   R   G   D   Y   K   W   Y   F
VH403                         ctc agc agc ctg aca tct gag gac tct gcg gtc tat ttc tgt gca aga ggg gac tat aaa tgg tac tt
                                                                           Y
AJ073939 Musmus IGHV1-64*01 F --- --- --- --- --- --- --- --- --- --- --- --- -a- --- --- ---

                              _________
                                 D   V   W   G   S   G   T   T   V   T   V   S   S
VH403                         c gat gtc tgg ggc tca ggg acc acg gtc acc gtc tcc tca
AJ073939 Musmus IGHV1-64*01 F
```

***(Figure S1 A, part 1)***

**VL 408**

```
                                 <-------------------------------------------  FR1 - IMGT  -----------------------------
                                 1              5                   10                  15                  20
                                  D   I   Q   M   T   Q   S   P   A   S   L   S   A   S   V   G   A   T   V   T   I   T   C
VL408                            gac att cag atg acc cag tct cca gct tcc ctg tct gca tct gtg gga gca act gtc acc atc aca tg
                                                                                  V                   E
AJ235956 Musmus IGKV12-46*01 F   --- --c --- --- --t --- --- --- --c --- --a --- -t- --- --- --- -a- --- --- --- --- --- --

                                 ------------>  _________________  CDR1 - IMGT  _____________  <---------------------------
                                       25                  30                  35                  40                  45
                                    R   T   S   E   N   I                                D   S   Y   L   A   W   Y   Q   Q   R
VL408                            t cga aca agt gag aat att ... ... ... ... ... ... gac agt tat tta gca tgg tat cag cag aga
                                        A                                                Y       N                       K
AJ235956 Musmus IGKV12-46*01 F   - --- g-- --- --- --- --- ... ... ... ... ... ... t-- --- a-- --- --- --- --- --- --- -a-

                                  FR2 - IMGT  ------------------------->  _____________  CDR2 - IMGT  _________  <---------
                                                 50                  55                  60                  65
                                  Q   G   K   S   P   Q   L   L   V   Y   A   A                               T   N   L   A
VL408                            cag gga aaa tct cct cag ctc ctg gtc tat gca gca ... ... ... ... ... ... ... aca aac tta gc
                                 
AJ235956 Musmus IGKV12-46*01 F   --- --- --- --- --- --- --- --- --- --- --t --- ... ... ... ... ... ... ... --- --- --- --

                                 -------------------------------------------------------------  FR3 - IMGT  ---------------
                                       70                  75                  80                  85                  90
                                    D   G   V   P       S   R   F   S   G   S   G           S   G   T   Q   Y   S   L   K
VL408                            a gat ggt gtg cca ... tca agg ttc agt ggc agt gga ... ... tca ggc aca cag tat tct ctc aag
                                 
AJ235956 Musmus IGKV12-46*01 F   - --- --- --- --- ... --- --- --- --- --- --- --- ... ... --- --- --- --- --- --c --- ---

                                 ------------------------------------------------------>  ___________  CDR3 -  IMGT  ________
                                                 95                  100                 104
                                  I   N   S   L   Q   S   E   D   V   A   R   Y   Y   C   Q   H   Y   S   T   T   P   W   T
VL408                            atc aac agc ctg cag tct gaa gat gtt gcg aga tat tac tgt caa cat tat tct act act ccg tgg ac
                                                                  F   G   S                           F   W   G           P
AJ235956 Musmus IGKV12-46*01 F   --- --- --- --- --- --- --- --- t-- -g- --t --- --- --- --- --- -t- -gg gg- --- --t cc


                                    F   G   G   G   T   Q   L   E   I   K   R
VL408                            g ttc ggt gga ggc acc cag ctg gaa ata aaa cgt

AJ235956 Musmus IGKV12-46*01 F
```

**VH 408**

```
                                 <-------------------------------------------  FR1 - IMGT  -----------------------------
                                 1              5                   10                  15                  20
                                  E   V   Q   L   Q   Q   S   G   P       E   L   V   K   P   G   A   S   V   K   I   S   C
VH408                            gag gtc cag ctg cag cag tct gga cct ... gag ctg gtg aag cct ggg gct tca gtg aag ata tcc tg
                                 
AC079181 Musmus IGHV1-42*01 F    --- --- --- --- --- --- --- --- --- ... --- --- --- --- --- --- --- --- --- --- --- --- --

                                 ------------>  _________________  CDR1 - IMGT  _____________  <---------------------------
                                       25                  30                  35                  40                  45
                                    K   A   S   G   Y   S   F                   T   G   Y   Y   M   N   W   V   K   Q   S
VH408                            c aag gct tct ggt tac tca ttc ... ... ... ... act ggc tac tac atg aac tgg gtg aaa caa agt
                                 
AC079181 Musmus IGHV1-42*01 F    - --- --- --- --- --- --- --- ... ... ... ... --- --- --- --- --- --- --- --- --- --g --- ---

                                  FR2 - IMGT  ------------------------->  _____________  CDR2 - IMGT  _________  <---------
                                                 50                  55                  60                  65
                                  P   E   K   S   L   E   W   I   G   E   M   S   P   S           T   G   R   T   T   Y   N
VH408                            cct gaa aag agc ctt gag tgg att gga gag atg agt cct agc ... ... act ggt cga act acc tac aa
                                                                          I   N                           G
AC079181 Musmus IGHV1-42*01 F    --- --- --- --- --- --- --- --- --- --- --t -a- --- --- ... ... --- --- g-t --- --- --- --

                                 -------------------------------------------------------------  FR3 - IMGT  ---------------
                                       70                  75                  80                  85                  90
                                    Q   N   F   K       A   K   A   T   L   T   V   D   Q   S   S   S   T   A   Y   M   Q
VH408                            t cag aat ttt aag ... gcc aag gcc aca ttg act gta gac caa tcc tcc agc aca gcc tac atg cag
                                        K                                           K
AC079181 Musmus IGHV1-42*01 F    c --- --g --c --- ... --- --- --- --- --- --- --- --- a-- --- --- --- --- --- --- --- ---

                                 ------------------------------------------------------>  ___________________________  CDR3
                                                 95                  100                 104
                                  L   K   S   L   T   S   E   D   S   A   V   Y   Y   C   A   R   S   V   P   L   T   T   L
VH408                            ctc aag agc ctg aca tct gag gac tct gca gtc tat tac tgt gca aga agt gtc ccc tta act acg tt
                                 
AC079181 Musmus IGHV1-42*01 F    --- --- --- --- --- --- --- --- --- --- --- --- --- --- --- ---

                                 -  IMGT  _________________________

                                    I   E   D   W   Y   F   D   V   W   G   T   G   T   T   V   T   V   S   S
VH408                            a ata gag gac tgg tac ttc gat gtc tgg ggc aca ggg acc acg gtc acc gtc tcc tca

AC079181 Musmus IGHV1-42*01 F
```

***(Figure S1 A, part 2)***

**VL 410**

```
                                 <--------------------------------------------- FR1 - IMGT ------------------------------
                                 1           5                   10                  15                  20
                                  Q   I   V   L   T   Q   S   P   A   I   M   S   A   S   P   G   E   K   V   T   M   T   C
VL410                            caa att gtt ctc acc cag tct cca gca atc atg tct gca tct cca ggg gag aag gtc acc atg acc tg
                                                                                                                 I   S
AJ231209 Musmus IGKV4-61*01 F    --- --- --- --- --- --- --- --- --- --- --- --- --- --- --- --- --- --- --- --- --a t-- --

                                 ------------> __________________ CDR1 - IMGT ______________ <---------------------------
                                       25                  30                  35                  40                  45
                                   S   A   S   S   S   V                               S   Y   M   Y   W   Y   H   Q   K
VL410                            c agt gcc agc tca agt gta ... ... ... ... ... ... ... agt tac atg tac tgg tac cat cag aag
                                                                                                               Q
AJ231209 Musmus IGKV4-61*01 F    - --- --- --- --- --- --- ... ... ... ... ... ... ... --- --- --- --- --- --- --g --- ---

                                  FR2 - IMGT -------------------------> ______________ CDR2 - IMGT __________ <---------
                                               50                  55                  60                  65
                                  P   G   S   S   P   K   P   W   I   Y   R   T                               S   N   L   A
VL410                            cca gga tcc tca ccc aaa ccc tgg att tat cgc aca ... ... ... ... ... ... ... tcc aac ctg gc

AJ231209 Musmus IGKV4-61*01 F    --- --- --- --c --- --- --- --- --- --- --- --- ... ... ... ... ... ... ... --- --- --- --

                                 ------------------------------------------------------------- FR3 - IMGT ---------------
                                       70                  75                  80                  85                  90
                                   S   G   V   P       A   R   F   S   G   S   G           S   G   T   S   Y   S   L   S
VL410                            t tct gga gtc cct ... gct cgc ttc agt ggc agt ggg ... ... tct ggg acc tct tac tct ctc tca
                                                                                                                       T
AJ231209 Musmus IGKV4-61*01 F    - --- --- --- --- ... --- --- --- --- --- --- --- ... ... --- --- --- --- --- --- --- a--

                                 ------------------------------------------------------> ___________ CDR3 -  IMGT ________
                                                 95                  100             104
                                  V   S   S   V   E   A   E   D   A   A   T   Y   Y   C   Q   Q   Y   N   S   H   P   M   T
VL410                            gtc agc agc gtg gag gcc gaa gat gct gcc act tat tac tgc cag cag tac aat agt cac ccc atg ac
                                  I           M                                                       H       Y       P
AJ231209 Musmus IGKV4-61*01 F    a-- --- --- a-- --- --t --- --- --- --- --- --- --- --- --- --- --t c-- --- t-- --a ccc -

                                 _

                                   F   G   G   G   T   K   L   E   I   K   R
VL410                            g ttc ggt gga ggc acc aag ttg gaa atc aaa cgg

AJ231209 Musmus IGKV4-61*01 F
```

**VH 410**

```
                                 <--------------------------------------------- FR1 - IMGT ------------------------------
                                 1           5                   10                  15                  20
                                  E   V   Q   L   V   E   S   G   G       G   L   V   Q   P   K   G   S   L   K   L   S   C
VH410                            gag gtg cag ctt gtt gag tct ggt gga ... gga ttg gtg cag cct aaa gga tca ttg aaa ctc tca tg

AC073561 Musmus IGHV10-3*01 F    --- --- --- --- --- --- --- --- --- ... --- --- --- --- --- --- --- --- --- --- --- --- --

                                 ------------> __________________ CDR1 - IMGT ______________ <---------------------------
                                       25                  30                  35                  40                  45
                                   A   A   S   G   F   T   F                   N   T   Y   A   M   H   W   V   R   Q   A
VH410                            t gcc gcc tct ggt ttc acc ttc ... ... ... ... aat acc tat gcc atg cac tgg gtc cgc cag gct

AC073561 Musmus IGHV10-3*01 F    - --- --- --- --- --- --- --- ... ... ... ... --- --- --- --- --- --- --- --- --- --- ---

                                  FR2 - IMGT -------------------------> ______________ CDR2 - IMGT __________ <---------
                                               50                  55                  60                  65
                                  P   G   K   G   L   E   W   V   A   R   I   R   S   K   S   N   K   Y   A   T   H   Y   A
VH410                            cca gga aag ggt ttg gaa tgg gtt gct cgc ata aga agt aaa agt aat aaa tat gca aca cat tat gc
                                                                                              S   N                   Y
AC073561 Musmus IGHV10-3*01 F    --- --- --- --- --- --- --- --- --- --- --- --- --- --- --- -g- --t --- --- --- t-- --- --

                                 ------------------------------------------------------------- FR3 - IMGT ---------------
                                       70                  75                  80                  85                  90
                                   D   S   V   K       D   R   F   T   I   S   R   D   D   S   Q   T   M   L   Y   L   Q
VH410                            c gat tca gtg aaa ... gac aga ttc acc atc tcc aga gat gat tca caa acc atg ctc tat ctg caa
                                                                                                   S
AC073561 Musmus IGHV10-3*01 F    - --- --- --- --- ... --- --- --- --- --- --- --- --- --- --- --- -g- --- --- --- --- ---

                                 ------------------------------------------------------> __________________________ CDR3 -
                                                 95                  100             104
                                  M   N   N   L   K   T   E   D   T   A   M   Y   Y   C   V   R   E   G   S   Y   Y   D   S
VH410                            atg aac aac ctg aaa act gag gac aca gcc atg tat tac tgt gtg aga gaa ggg agt tac tac gat ag

AC073561 Musmus IGHV10-3*01 F    --- --- --- --- --- --- --- --- --- --- --- --- --- --- --- --- -

                                  IMGT ______________________

                                   S   Y   G   A   M   D   Y   W   G   Q   G   T   S   V   T   V   S   S
VH410                            t agc tac ggt gct atg gac tac tgg ggt caa gga acc tca gtc acc gtc tcc tca

AC073561 Musmus IGHV10-3*01 F
```

***(Figure S1 A, part 3)***

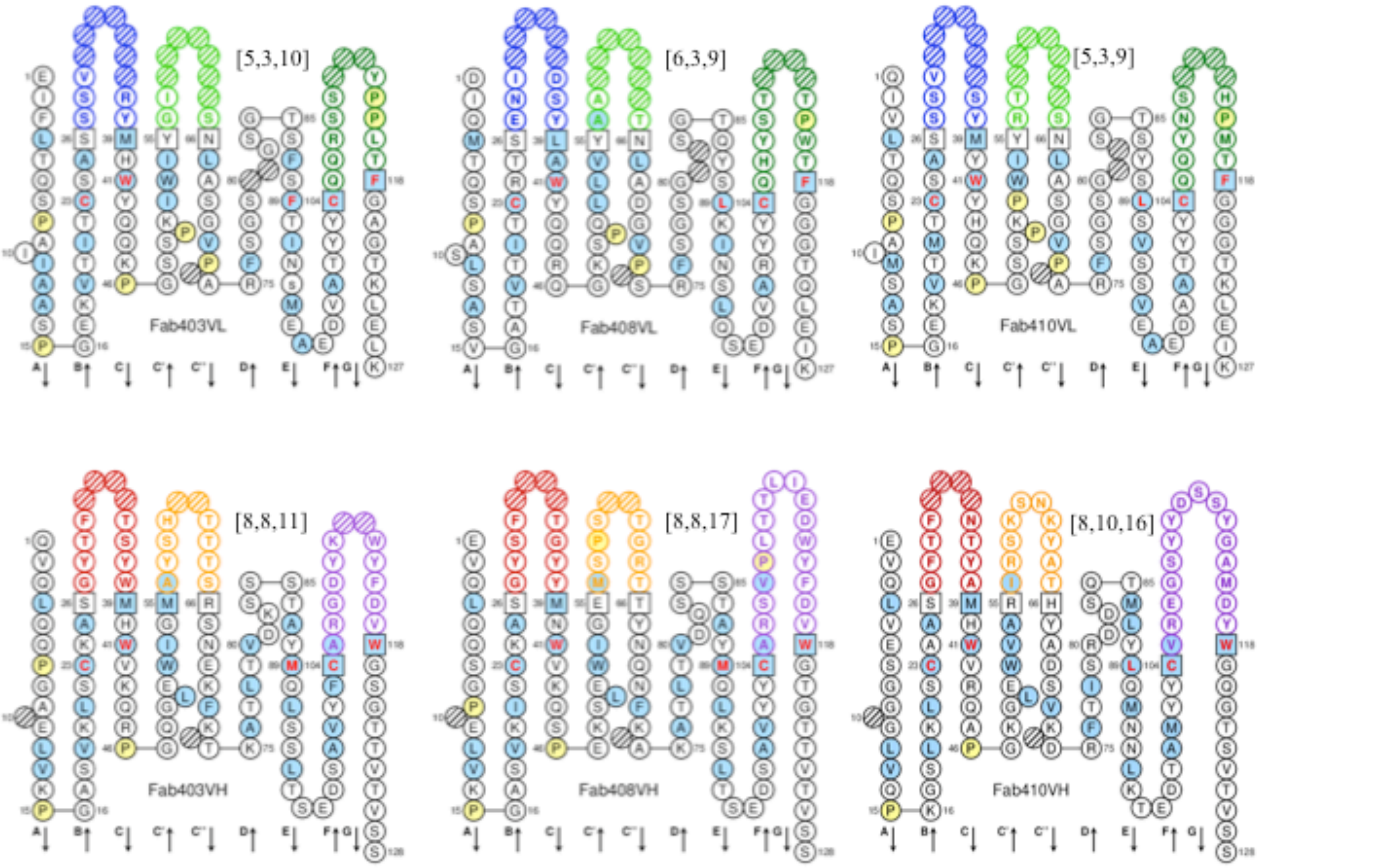


***(Figure S1 B)***

**Figure S1. The variable regions of Elec403, Elec408 and Elec410. (A, parts 1-3)** Alignments of the rearranged nucleotide sequences of the VL and VH regions with the IMGT/V-QUEST reference directory sets of germline V-REGION alleles (only this part of the variable region, not the entire variable region, is shown). *Bold red letters* denote the modified amino acid residues and nucleotides in the antibody compared to the germline. *Dashes* indicate identical nucleotides. *Dots* indicate gaps according to the IMGT unique numbering. **(B)** IMGT canonical representation of the Fab403, Fab 408 and Fab410 variable domains. CDR-L1, CDR-L2, CDR-L3 are displayed in blue, light green, dark green, and CDR-H1, CDR-H2, CDR-H3 in red, orange, purple, respectively. The five conserved residues/positions of the VL and VH domains are displayed with red bold letters. Anchor positions are squared. Gaps in the IMGT numbering are hatched. Pro residues are shown on a yellow background. Arrows indicate the theoretical main ß-strands and their direction. The greater lengths of CDRs H3 in Fab408 and Fab410, and of CDR-H2 in Fab410, compared with their counterparts in the other Fabs, is evident. Theoretical pI values for the Fab403 CDRs are: 8.46 (L1), 5.52 (L2), 8.22 (L3) and 5.24 (H1), 8.52 (H2), 5.95 (H3). For the Fab408 CDRs they are: 3.67 (L1), 5.52 (L2), 6.73 (L3) and 5.25 (H1), 6.10 (H2), 4.03 (H3). And for the Fab410 CDRs they are 5.24 (L1), 8.75 (L2), 6.73 (L3) and 5.24 (H1), 11.10 (H2), 4.03 (H3). (Figure made with the *IMGT/Collier-de-Perles* tool using the 50% hydrophobic position option.)

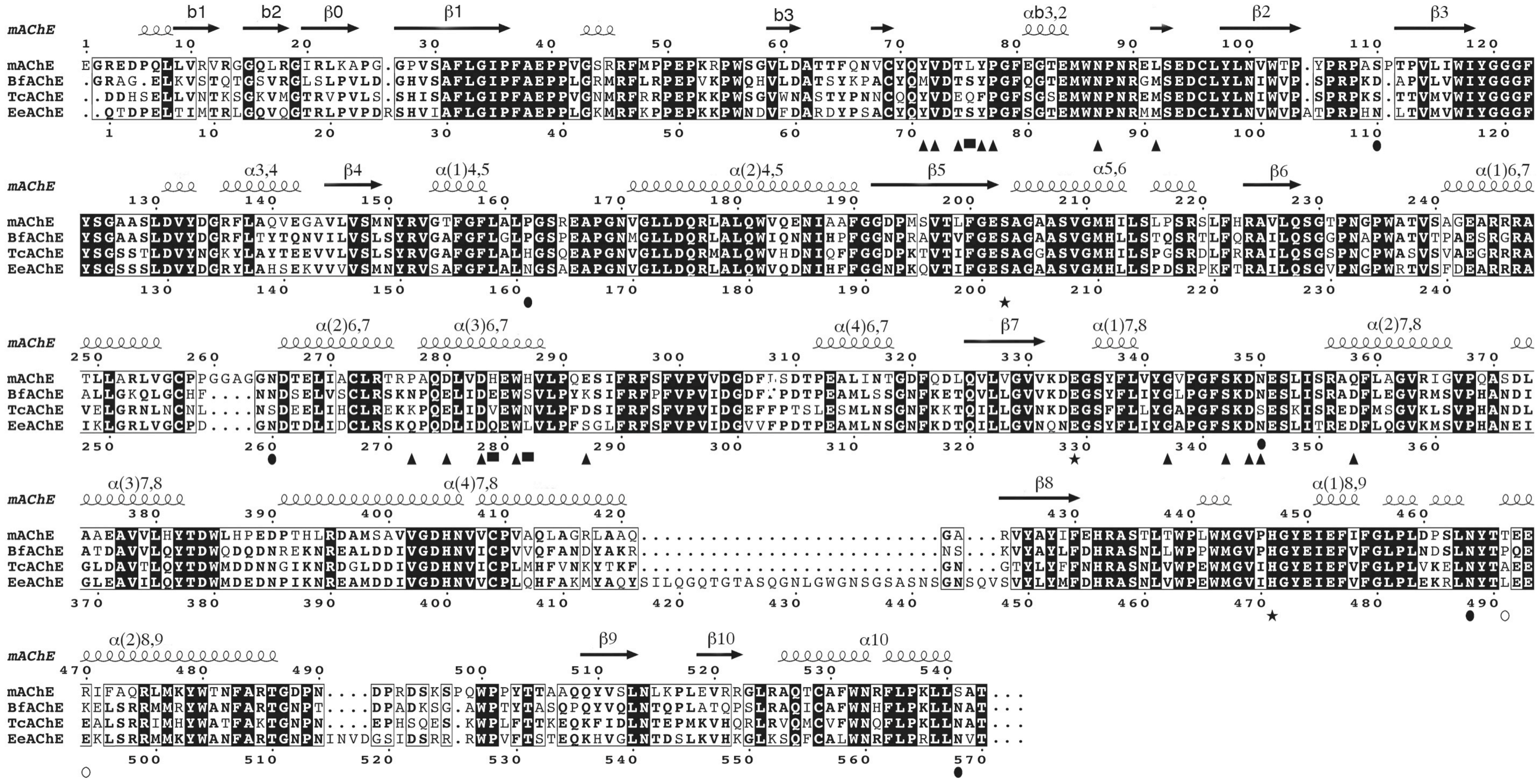

**Figure S2. Sequence alignment of the AChE species cited in this study.** The sequences of the EeAChE, TcAChE, BfAChE and mAChE subunits are displayed. The residue numbering displayed below the alignment is that of EeAChE, while the residue numbering and secondary structure elements displayed above the alignment are those of mAChE. Conserved residues are shown on a *black* background and non-conserved residues on a *white* background. The symbols below the alignment point to: the catalytic triad residues (stars); EeAChE Asn residues that belong to consensus N-glycosylation sequences (black dots) [48]; EeAChE residues whose substitution by rat AChE residues abolished Elec403 binding (Ser75, Gln279, Leu282; black squares), Elec410 binding (Ser75) or Elec408 binding (Leu491, Glu494; white dots) [33]; and EeAChE residues found to be buried at the interface of the theoretical Fab403-EeAChE complex (triangles).

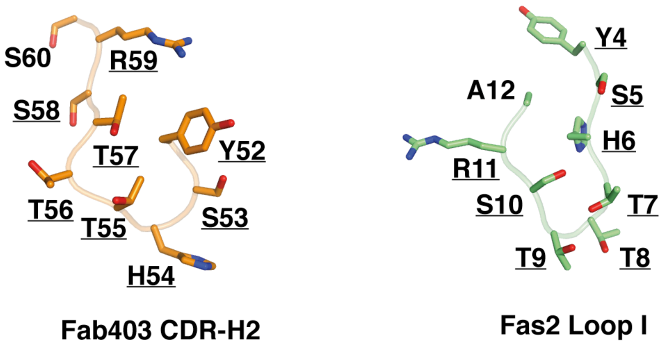


**Figure S3. Structural comparison of Fab403 CDR-H2 and Fas2 loop I.** Side by side views of Fab403 CDR-H2 (AYSHTTTSRS) and Fas2 loop I (YSHTTTSRAILTN) showing the distinctive Cα conformations and side chain repartitions despite their high sequence homology. The respective loop conformations are stabilized by intra-loop interactions. Fas2 residue Arg11 protrudes into the solvent and is well positioned at the loop I edge for interaction with AChE, whereas Fab403 residue Arg59 lies at the CDR base with limited accessibility for partnering.
